# The risk and prognostic factors for liver metastases in esophageal cancer patients: A large‐cohort based study

**DOI:** 10.1111/1759-7714.14642

**Published:** 2022-09-28

**Authors:** Peng Luo, Xiufeng Wei, Chen Liu, Xiankai Chen, Yafan Yang, Ruixiang Zhang, Xiaozheng Kang, Jianjun Qin, Xiuzhu Qi, Yin Li

**Affiliations:** ^1^ Department of Thoracic Surgery, National Cancer Center, National Clinical Research Center for Cancer, Cancer Hospital Chinese Academy of Medical Sciences and Peking Union Medical College Beijing China; ^2^ Department of Thoracic Surgery, Beijing Chuiyangliu Hospital Chuiyangliu Hospital Affiliated to Tsinghua University Beijing China; ^3^ Department of Ophthalmology, Shanghai Changhai Hospital Naval Military Medical University Shanghai China; ^4^ Department of Ultrasound Fudan University Shanghai Cancer Center Shanghai China; ^5^ Department of Oncology, Shanghai Medical College Fudan University Shanghai China

**Keywords:** esophageal cancer, introduction, liver metastases, nomogram, prognosis, risk factors

## Abstract

**Background:**

This retrospective study aimed to explore risk factors for liver metastases (LiM) in patients with esophageal cancer (EC) and to identify prognostic factors in patients initially diagnosed with LiM.

**Methods:**

A total of 28 654 EC patients were retrieved from the Surveillance, Epidemiology and End Results (SEER) database from 2010 to 2018. A multivariate logistic regression model was utilized to identify risk factors for LiM. A Cox regression model was used to identify prognostic factors for patients with LiM.

**Results:**

Of 28 654 EC patients, 4062 (14.2%) had LiM at diagnosis. The median overall survival (OS) for patients with and without LiM was 6.00 (95% CI: 5.70–6.30) months and 15.00 (95% CI: 14.64–15.36) months, respectively. Variables significantly associated with LiM included gender, age, tumor site, histology, tumor grade, tumor size, clinical T stage, clinical N stage, bone metastases (BoM), brain metastases (BrM) and lung metastases (LuM). Variables independently predicting survival for EC patients with LiM were age, histology, tumor grade, BoM, BrM, LuM, and chemotherapy. A risk prediction model and two survival prediction models were then constructed revealing satisfactory predictive accuracy.

**Conclusions:**

Based on the largest known cohort of EC, independent predictors of LiM and prognostic indicators of survival for patients with LiM were identified. Two models for predicting survival as well as a risk prediction model were developed with robust predictive accuracy.

## Graphical abstract

The risk factors for liver metastases in esophageal cancer.

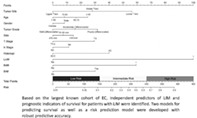

According to epidemiological estimates, there is a predicted 19 260 new instances of esophageal cancer (EC) yearly in the US, ranking it as the eighth most prevalent malignancy in the world.^1^, ^2^ EC is a highly lethal malignancy, with a propensity for regional lymph node metastases and distant metastases (DM), resulting in a 5‐year overall survival (OS) of only 30%–40%.^3^

Given the relatively low prevalence of EC in western nations, screening examinations are not routinely performed.^4^ As a result, many EC patients are diagnosed after the disease has metastasized. In the United Kingdom, 37%–42% of newly diagnosed EC patients present with DM.^4^, ^5^ Metastatic EC (mEC) is treatable but not curable, with the therapeutic goals being palliative care and extending survival. Notably, the 5‐year overall survival rate of mEC stands at only 5%, with a median OS of 8–10 months.^6^, ^7^

According to existing literature, the liver is the most common organ affected by distant metastasis, accounting for 32.4%–56.0%.^8^, ^9^ Early diagnosis and intervention for liver metastases (LiM) are essential to improve patient outcomes. Several studies previously revealed the metastatic patterns and clinical prognosis of mEC patients.^8^, ^9^, ^10^ However, risk factors and prognostic factors specifically for LiM have not been fully explored. Therefore, predictive models for LiM probability and prognosis of EC patients with LiM are urgently needed to facilitate metastatic screening and predict clinical outcomes.

This study aimed to develop a novel risk prediction model for predicting the likelihood of LiM in newly diagnosed EC patients as well as a survival prediction model for predicting the prognosis of EC patients with LiM using the Surveillance, Epidemiology, and End Results (SEER) database.

## METHODS

### Patient selection and data collection

Eligible patients diagnosed with EC from the SEER database were selected for this study. Patients diagnosed from 2010 to 2018 were screened according to histology codes 8050–8089 (squamous cell neoplasms) and 8140–8389 (adenomas and adenocarcinomas). The exclusion criteria were as follows^1^: T0, or Tis disease,^2^ liver metastases of unknown status,^3^ lacking survival time data or survival time <1 month,^4^ inadequate tumor data. Parameters of interest, including race, gender, age at diagnosis, histology, the American Joint Commission on Cancer (AJCC) tumor‐node‐metastasis (TNM) staging system, tumor size, grade, and treatment were obtained from the database. The primary tumor site was classified into the upper esophagus, middle esophagus, and lower esophagus. The cervical and abdominal esophagus were considered as the upper and lower esophagus, respectively. Tumor sites of overlapping and unspecified thoracic esophagus were counted as the primary site of unknown. Patients with records of esophagectomy were deemed to have received surgery.

### Statistical analysis

Demographic information and clinicopathological characteristics were summarized and presented using counts and percentages. Univariable and multivariable logistic regression models were used to identify predictors and build the risk model for EC patients with LiM. Univariable and multivariable Cox regression models were adopted to identify independent prognostic factors and build the risk model for OS and cancer‐specific survival (CSS). The missing values were dealt with in multivariable analyses using the multiple imputation method. Nomograms were established their accuracies were validated by receiver operating characteristic (ROC) curves and calibration slopes. All tests were two‐sided, with the statistical significance set at *p* < 0.05. All statistical analyses were conducted using R software 3.5.2 (R Foundation for Statistical Computing, Vienna, Austria).

## RESULTS

### Baseline clinical characteristics

A total of 28 654 eligible patients diagnosed with EC from 2010 to 2018 were obtained from the SEER database, with 4062 (14.2%) cases having LiM at diagnosis. Male patients accounted for 78.6% (*n* = 22 533). The median age was 67, with the age group of 60–70 accounting for 33.9% (*n* = 9707). White race was ranked first in terms of incidence (*n* = 24 051, 83.9%), followed by black (*n* = 2896, 10.1%) and other races (*n* = 1707, 6.0%). The lower esophagus was the most common primary tumor site, accounting for 61.5% (*n* = 17 631). There were 18 837 (65.7%) patients with adenocarcinoma and 9817 (34.3%) with squamous cell carcinoma. Histologically poorly differentiated EC accounted for 38.9% (*n* = 11 160), followed by moderately differentiated (*n* = 10 169, 35.5%) and well‐differentiated EC (*n* = 1510, 5.3%). A total of 9247 (32.3%) had tumor sizes ranging from 15 to 50 mm. There were 9541 (33.3%) patients with T3 disease, 6971 (24.3%) with T1, 2940 (10.3%) with T2, and 2774 (9.7%) with T4. Regional lymph node metastases were diagnosed clinically in 14 330 (50.0%) patients. Concurrent metastases of bone, brain and lung were present in 7.4% (*n* = 2125), 1.7% (*n* = 481), and 8.7% (*n* = 2506) of EC patients, respectively. Surgery, radiotherapy and chemotherapy were adopted in 6247 (21.8%), 11 230 (39.2%) and 19 371 (67.6%) cases, respectively. Demographic information and tumor characteristics are listed in Table 1.

**TABLE 1:** Clinicopathological variables of 28 654 esophageal cancer patients diagnosed from 2010 to 2018

Variables	Total (*N* = 28 654)	Without LiM (*N* = 24 592)	With LiM (*N* = 4062)
Gender			
Male	22 533 (78.6%)	19 055 (77.5%)	3478 (85.6%)
Female	6121 (21.4%)	5537 (22.5%)	584 (14.4%)
Age (years)			
≤60	7906 (27.6%)	6505 (26.5%)	1401 (34.5%)
60–70	9707 (33.9%)	8316 (33.8%)	1391 (34.2%)
70–80	7170 (25.0%)	6271 (25.5%)	899 (22.1%)
>80	3871 (13.5%)	3500 (14.2%)	371 (9.1%)
Race			
White	24 051 (83.9%)	20 493 (83.3%)	3558 (87.6%)
Black	2896 (10.1%)	2573 (10.5%)	323 (8.0%)
Other	1707 (6.0%)	1526 (6.2%)	181 (4.5%)
Tumor site			
Upp	2209 (7.7%)	2112 (8.6%)	97 (2.4%)
Mid	4451 (15.5%)	4123 (16.8%)	328 (8.1%)
Low	17 631 (61.5%)	14 664 (59.6%)	2967 (73.0%)
Unknown	4363 (15.2%)	3693 (15%)	670 (16.5%)
Histology			
AC	18 837 (65.7%)	15 517 (63.1%)	3320 (81.7%)
SCC	9817 (34.3%)	9075 (36.9%)	742 (18.3%)
Tumor grade			
I	1510 (5.3%)	1409 (5.7%)	101 (2.5%)
II	10 169 (35.5%)	8905 (36.2%)	1219 (30.0%)
III	11 160 (38.9%)	9194 (37.4%)	1966 (48.4%)
Unknown	5815 (20.3%)	5039 (20.5%)	776 (19.1%)
Tumor size (mm)			
≤15	1996 (7.0%)	1920 (7.8%)	76 (1.9%)
15–50	9247 (32.3%)	8176 (33.2%)	1071 (26.4%)
>50	6343 (22.1%)	5378 (21.9%)	965 (23.8%)
Unknown	11 068 (38.6%)	9118 (37.1%)	1950 (48%)
T stage			
T1	6971 (24.3%)	6192 (25.2%)	779 (19.2%)
T2	2940 (10.3%)	2786 (11.3%)	154 (3.8%)
T3	9541 (33.3%)	8895 (36.2%)	646 (15.9%)
T4	2774 (9.7%)	2197 (8.9%)	577 (14.2%)
Unknown	6428 (22.4%)	4522 (18.4%)	1906 (46.9%)
Regional lymph node			
N0	12 139 (42.4%)	10 964 (44.6%)	1175 (28.9%)
N+	14 330 (50.0%)	12 065 (49.1%)	2265 (55.8%)
Unknown	2185 (7.6%)	1563 (6.4%)	622 (15.3%)
BoM			
Yes	2125 (7.4%)	1290 (5.2%)	835 (20.6%)
No/unknown	26 529 (92.6%)	23 302 (94.8%)	3227 (79.4%)
BrM			
Yes	481 (1.7%)	299 (1.2%)	182 (4.5%)
No/unknown	28 173 (98.3%)	24 293 (98.8%)	3880 (95.5%)
LuM			
Yes	2506 (8.7%)	1363 (5.5%)	1143 (28.1%)
No	26 148 (91.3%)	23 229 (94%)	2919 (68.8%)
Surgery			
Yes	6247 (21.8%)	6215 (25.3%)	31 (0.8%)
No/unknown	22 407 (78.2%)	18 377 (74.7%)	4031 (99.2%)
Radiotherapy			
Yes	11 230 (39.2%)	15 997 (65.0%)	1427 (35.1%)
No/unknown	17 424 (60.8%)	8595 (35.0%)	2635 (64.9%)
Chemotherapy			
Yes	19 371 (67.6%)	16 505 (67.1%)	2866 (70.6%)
No/unknown	9283 (32.4%)	8087 (32.9%)	1196 (29.4%)

Abbreviations: AC, adenocarcinoma; BoM, bone metastases; BrM, brain metastases; Grade I, well differentiated; Grade II, moderately differentiated; Grade III, poorly differentiated; LiM, liver metastases, LuM, lung metastases; Low, lower esophagus; Mid, middle esophagus; N+, clinically diagnosed metastatic regional lymph node; SCC, squamous cell carcinoma; Upp, upper esophagus.

### Risk factors for developing liver metastases

**FIGURE 1:**
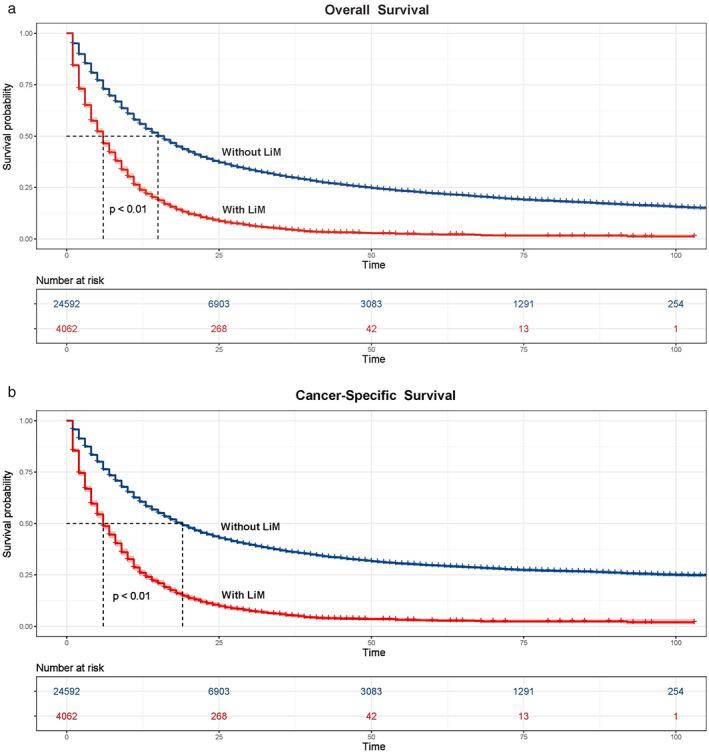
Kaplan–Meier curves and risk tables of (a) overall survival and (b) cancer‐specific survival for esophagus cancer patients with and without brain metastases. LiM, liver metastases

According to univariate and multivariate analyses, several variables were determined as independent risk factors for LiM, including gender, age, tumor site, histology, tumor grade, tumor size, T stage, regional lymph node, BoM, BrM and LuM. The details are provided in Table 2. In terms of gender, the male sex was a higher risk factor for LiM (OR = 1.17, 95% CI: 1.06–1.29, *p* < 0.01). Additionally, older age at diagnosis was negatively associated with the incidence of LiM, while patients older than 80 had the lowest risks (OR = 0.62, 95% CI: 0.54–0.70, *p* < 0.01). As for the tumor site, an increasing risk of LiM was detected as the tumor site got lower. A tumor of the lower esophagus was 2.56 times more likely than that of the upper esophagus to have LiM (95% CI: 2.11–3.12, *p* < 0.01). Histologically, LiM was more common in patients with AC histology (OR = 2.06, 95% CI: 1.84–2.31, *p* < 0.01). In terms of tumor grade, a higher tumor grade was significantly associated with a higher incidence of LiM (*p* < 0.01). Primary tumor of T4 possessed a higher risk of LiM compared to that of T1‐3 (OR = 1.70, 95% CI: 1.54–1.88, *p* < 0.01). In addition, LiM was more common in patients with regional lymph node metastases (OR = 1.21, 95% CI: 1.12–1.31, *p* < 0.01). Furthermore, BoM (OR = 2.89, 95% CI: 2.60–3.20, *p* < 0.01), BrM (OR = 1.72, 95% CI: 1.39–2.13, *p* < 0.01) and LuM (OR = 5.98, 95% CI: 5.43–6.59, *p* < 0.01) were independently associated with a higher incidence of LiM (Figure 1).

**TABLE 2:** Univariable and multivariable logistic regression analyses for risk factors of liver metastases in esophageal cancer patients

	Univariable		Multivariable	
Variables	OR (95% CI)	*p*‐value	OR (95% CI)	*p*‐value
Gender				
Male vs. female	1.73 (1.58–1.90)	<0.01	1.17 (1.05–1.29)	<0.01
Age (years)				
≤60	(Reference)		(Reference)	
60–70	0.78 (0.72–0.84)	<0.01	0.82 (0.75–0.90)	<0.01
70–80	0.67 (0.61–0.73)	<0.01	0.77 (0.69–0.85)	<0.01
>80	0.49 (0.44–0.56)	<0.01	0.62 (0.54–0.70)	<0.01
Race				
White	(Reference)			
Black	0.72 (0.64–0.82)	<0.01	1.06 (0.92–1.23)	0.409
Other	0.68 (0.58–0.80)	<0.01	0.91 (0.77–1.09)	0.317
Tumor site				
Upp	(Reference)		(Reference)	
Mid	1.58 (1.31–1.91)	<0.01	1.47 (1.20–1.80)	<0.01
Low	3.42 (2.89–4.04)	<0.01	2.56 (2.11–3.12)	<0.01
Histology				
AC vs. SCC	2.62 (2.41–2.85)	<0.01	2.06 (1.84–2.31)	<0.01
Tumor grade				
I	(Reference)		(Reference)	
II	1.49 (1.25–1.76)	<0.01	1.43 (1.19–1.72)	<0.01
III	2.29 (1.93–2.72)	<0.01	1.86 (1.55–2.24)	<0.01
Tumor size (mm)				
≤15	(Reference)		(Reference)	
15–50	1.69 (1.48–1.93)	<0.01	1.46 (1.27–1.69)	<0.01
>50	2.18 (1.90–2.49)	<0.01	1.52 (1.31–1.76)	<0.01
T stage				
T4 vs. T1‐3	1.88 (1.72–2.05)	<0.01	1.70 (1.54–1.88)	<0.01
Regional lymph node				
N+ vs. N0	1.67 (1.56–1.79)	<0.01	1.21 (1.12–1.31)	<0.01
BoM				
Yes vs. no	4.67 (4.25–5.14)	<0.01	2.89 (2.60–3.20)	<0.01
BrM				
Yes vs. no	3.81 (3.16–4.60)	<0.01	1.72 (1.39–2.13)	<0.01
LuM				
Yes vs. no	6.67 (6.11–7.28)	<0.01	5.98 (5.43–6.59)	<0.01

### Performance and validation of the nomogram for predicting liver metastases in EC patients

**FIGURE 2:**
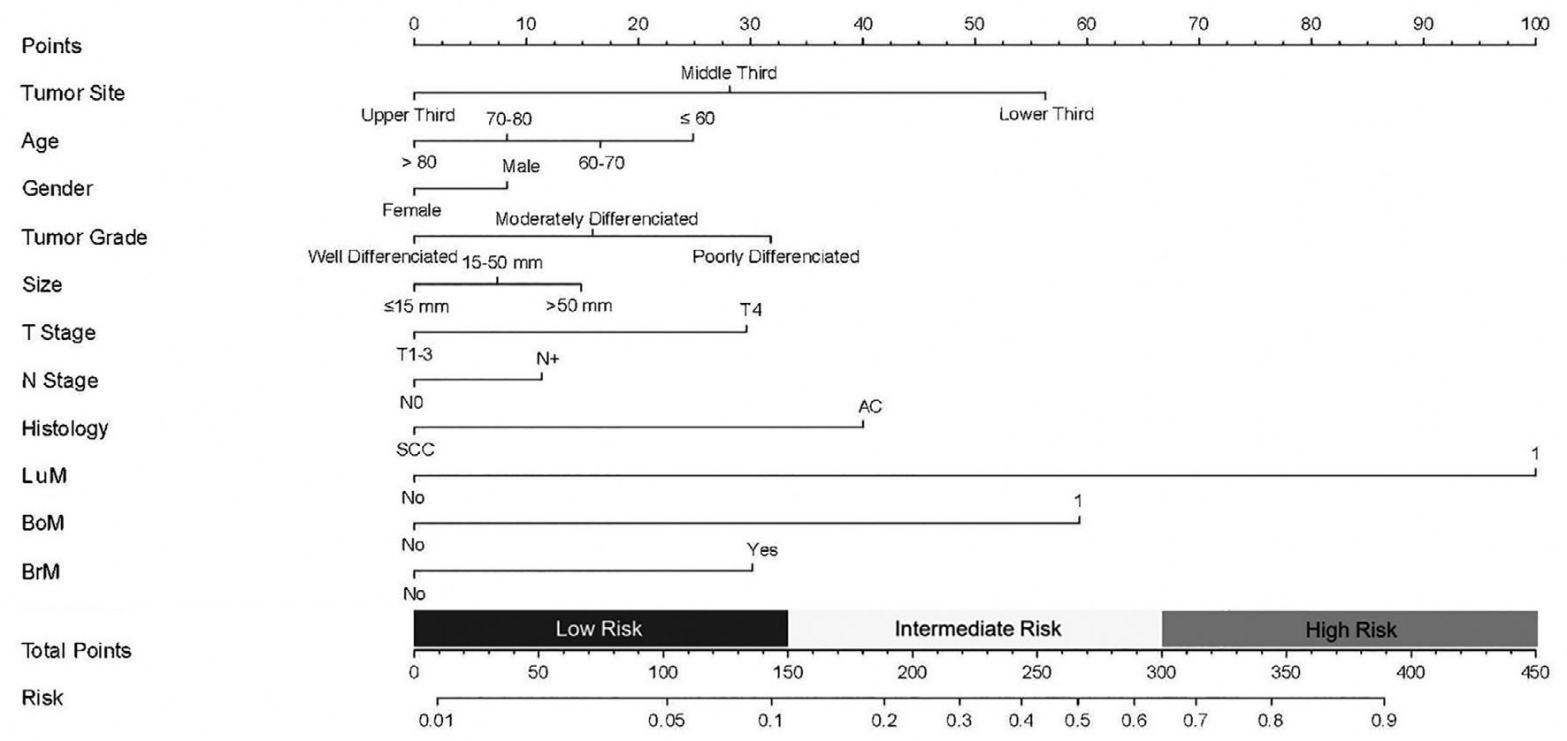
The nomogram for predicting the likelihood of liver metastasis in esophageal cancer patients

A nomogram was developed based on the risk factors identified to affect the probability of LiM in EC patients (Figure 2). The score of each integrated variable can be added together to determine each person's specific risk of LiM. The calibration slope and ROC curve were adopted to test the nomogram's accuracy. The calibration curve revealed a great consistency between the prediction of the model and the actual LiM probability (Supplementary Figure 1A). According to the ROC curve, the area under the curve (AUC) was 0.762, suggesting a satisfactory predictive accuracy (Supplementary Figure 1B).

### Survival and prognostic factors for esophageal cancer patients with liver metastases

The median OS of patients with and without LiM was 6.00 (95% CI: 5.70–6.30) months and 15.00 (95% CI: 14.64–15.36) months, respectively (Figure 1(a)). The median CSS of patients with and without LiM were 6.00 (95% CI: 5.69–6.31) months and 19.00 (95% CI: 18.47–19.53) months, respectively (Figure 1(b)). The 6‐month OS, 12‐month OS, and 18‐month OS were 46.4, 23.8, and 14.3%, respectively, while the 6‐month CSS, 12‐month CSS, and 18‐month CSS were 48.8, 26.0, and 16.0%, respectively. According to univariate analyses, age, race, tumor site, histology, tumor grade, tumor size, BoM, BrM, LuM, and chemotherapy were related to OS and CSS. A multivariate Cox regression model further revealed that age, histology, tumor grade, BoM, BrM, LuM, and chemotherapy remained statistically significant as predictors for OS and CSS. The details are summarized in Table 3. In terms of age, patients older than 70 showed decreased OS (HR = 1.16, 95% CI: 1.08–1.25, *p* < 0.01) and CSS (HR = 1.15, 95% CI: 1.06–1.23, *p* < 0.01). As for histology, favorable OS (HR = 0.80, 95% CI: 0.72–0.88, *p* < 0.01) and CSS (HR = 0.79, 95% CI: 0.72–0.88, *p* < 0.01) were observed in patients with the AC histology. Moreover, histologically poorly differentiated EC had a worse OS (HR = 1.17, 95% CI: 1.09–1.25, *p* < 0.01) and CSS (HR = 1.17, 95% CI: 1.09–1.26, *p* < 0.01) compared to moderately differentiated and well‐differentiated EC. With regard to other metastatic sites, patients with coexistent BoM showed significantly shortened OS (HR = 1.40, 95% CI: 1.30–1.53, *p* < 0.01) and CSS (HR = 1.38, 95% CI: 1.27–1.50, *p* < 0.01). Similar results were also found in patients with BrM (OS, HR = 1.33, 95% CI: 1.13–1.56, *p* < 0.01; CSS, HR = 1.33, 95% CI: 1.13–1.57, *p* < 0.01) and LuM (OS, HR = 1.17, 95% CI: 1.09–1.26, *p* < 0.01; CSS, HR = 1.20, 95% CI: 1.11–1.29, *p* < 0.01). In terms of treatment, patients that received chemotherapy had markedly better OS (HR = 0.34, 95% CI: 0.32–0.37, *p* < 0.01) and CSS (HR = 0.35, 95% CI: 0.32–0.38, *p* < 0.01). Variables including gender, race, tumor site, tumor size, T stage, regional lymph node, radiotherapy and surgery failed to reach statistical significance (*p* > 0.05).

**TABLE 3:** Univariable and multivariable cox regression analyses of prognostic factors for esophageal cancer patients with liver metastases

	OS				CSS			
	Univariable		Multivariable		Univariable		Multivariable	
Variables	HR (95% CI)	*p*‐value	HR (95% CI)	*p*‐value	HR (95% CI)	*p*‐value	HR (95% CI)	*p*‐value
Gender								
Male vs. female	1.02 (0.93–1.12)	0.643	–	–	1.02 (0.93–1.12)	0.700	–	–
Age (years)								
>70 vs. ≤70	1.27 (1.18–1.36)	<0.01	1.16 (1.08–1.25)	<0.01	1.25 (1.16–1.34)	<0.01	1.15 (1.06–1.23)	<0.01
Race								
White	(Reference)				(Reference)			
Black	1.25 (1.11–1.41)	<0.01	1.03 (0.90–1.17)	0.680	1.24 (1.10–1.41)	<0.01	1.02 (0.89–1.16)	0.815
Other	1.01 (0.86–1.19)	0.910	0.90 (0.76–1.06)	0.197	0.99 (0.84–1.18)	0.939	0.88 (0.74–1.04)	0.142
Tumor site								
Upp	(Reference)				(Reference)			
Mid	0.81 (0.67–0.98)	<0.05	0.90 (0.74–1.09)	0.281	0.83 (0.68–1.01)	0.067	0.92 (0.75–1.12)	0.410
Low	0.65 (0.55–0.78)	<0.01	0.89 (0.74–1.07)	0.226	0.66 (0.55–0.79)	<0.01	0.90 (0.75–1.10)	0.302
Histology								
AC vs. SCC	0.72 (0.66–0.78)	<0.01	0.80 (0.72–0.88)	<0.01	0.72 (0.66–0.78)	<0.01	0.79 (0.72–0.88)	<0.01
Tumor grade								
III vs. I/II	1.17 (1.09–1.25)	<0.01	1.17 (1.09–1.25)	<0.01	1.17 (1.09–1.25)	<0.01	1.17 (1.09–1.26)	<0.01
Tumor size (mm)								
≤50 vs. >50	1.08 (1.01–1.15)	<0.05	1.06 (1.00–1.14)	0.067	1.08 (1.01–1.16)	<0.05	1.06 (0.99–1.14)	0.078
T stage								
T4 vs. T1–3	1.06 (0.97–1.15)	0.189	–	–	1.07 (0.98–1.16)	0.126	–	–
Regional lymph node								
N+ vs. N0	0.94 (0.88–1.01)	0.089	‐	‐	0.96 (0.89–1.03)	0.256	‐	‐
BoM								
Yes vs. no	1.42 (1.31–1.54)	<0.01	1.41 (1.30–1.53)	<0.01	1.39 (1.28–1.51)	<0.01	1.38 (1.27–1.50)	<0.01
BrM								
Yes vs. no	1.42 (1.22–1.66)	<0.01	1.33 (1.13–1.56)	<0.01	1.43 (1.22–1.68)	<0.01	1.33 (1.13–1.57)	<0.01
LuM								
Yes vs. no	1.25 (1.16–1.35)	<0.01	1.17 (1.09–1.26)	<0.01	1.27 (1.18–1.37)	<0.01	1.20 (1.11–1.29)	<0.01
Surgery								
Yes vs. no/unknown	1.19 (0.83–1.70)	0.352	–	–	1.18 (0.81–1.70)	0.396	–	–
Radiotherapy								
Yes vs. no/unknown	1.07 (1.00–1.14)	0.066	–	–	1.06 (0.99–1.14)	0.082	–	–
Chemotherapy								
Yes vs. no/unknown	0.34 (0.31–0.36)	<0.01	0.34 (0.32–0.37)	<0.01	0.34 (0.32–0.37)	<0.01	0.35 (0.32–0.38)	<0.01

### Performance and validation of the nomogram for the survival of EC patients with liver metastases

**FIGURE 3:**
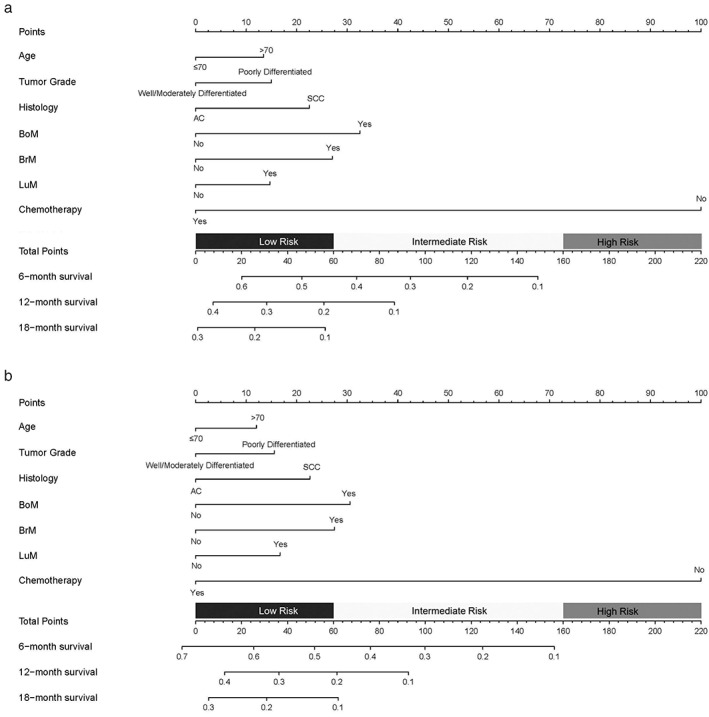
The nomogram for predicting 6‐, 12‐, and 18‐month (a) overall survival and (b) cancer‐specific survival of esophageal cancer patients with liver metastases

Next, two nomograms were constructed based on the independent OS (Figure 3(a)) and CSS (Figure 3(b)) prognostic factors identified in EC patients with LiM. By adding the outcomes of each included variable, the OS and CSS for each individual may be calculated accurately. Furthermore, the calibration slope and ROC curve validated the predictive accuracy of the two nomograms. The calibration curves showed a great consistency between calculated OS and actual OS (Supplementary Figure 2A,C,E), and a similar result was also observed for CSS (Supplementary Figure 2B,D,F). According to the ROC curve, all the AUCs were >0.73 for OS (Supplementary Figure 2G) and CSS (Supplementary Figure 2H), suggesting excellent predictive accuracy. ROC curves showed that our model had favorable efficiency for both OS and CSS compared to the eighth AJCC staging (AUC: 6‐month OS 0.770 vs. 0.594, 12‐month OS 0.745 vs. 0.557, 18‐month OS 0.742 vs. 0.557; 6‐month CSS 0.768 vs. 0.560, 12‐month DSS 0.739 vs. 0.567, 18‐month DSS 0.736 vs. 0.540). The ROC of the eighth AJCC staging curve is presented in Supplementary Figure 3.

## DISCUSSION

Esophageal cancer is a highly lethal malignancy, with the incidence of distant metastases in newly diagnosed EC patients being as high as 42% and the liver being the most commonly affected organ.^4^, ^5^, ^9^ The management of mEC requires a multimodality approach and remains challenging to date. Identifying high‐risk populations and early diagnosis of LiM in EC could facilitate treatment decision‐making, especially in avoiding futile radical surgery. Importantly, prognosis evaluation and the choice of a personalized treatment may benefit greatly from a prognostic prediction model.

Existing studies focusing on risk factors in EC patients with LiM are currently lacking.^9^, ^11^ Tang et al. previously constructed a nomogram to predict the survival of patients with metastatic EC.^12^ However, the study incorporated all metastatic sites, and a risk predicting nomogram for distant metastasis was not explored. Meanwhile, Cheng et al. established models predicting EC patient risk and survival, specifically for BrM.^13^ Additionally, Guo et al. described the characteristics and explored risk factors and prognostic factors for EC patients with LuM particularly but did not develop predictive tools.^14^ As the most common metastatic organ, a comprehensive study aiming specifically at EC patients with LiM is of great clinical importance. To the best of our knowledge, this study is the first study to explore both risk and prognostic factors for EC patients with LiM at diagnosis.

In the present cohort study, the incidence of LiM in initially diagnosed EC patients was 14.2%. In contrast, Verstegen et al. and Quint et al. previously reported a LiM rate of 8.9 and 5.2%, respectively,^8^, ^10^ which is markedly lower than our results. Interestingly, Verstegen et al. retrieved cases from 1990 to 2017, while Quint et al. analyzed a cohort from 1982 to 1993. However, in our study, only patients diagnosed from 2010 to 2018 were encompassed. Notably, new advances in imaging might have increased the likelihood of detecting early LiM in EC patients. Moreover, the sample size of our cohort was much larger than that of Verstegen et al. (28 654 vs. 1686) and Quint et al. (28 654 vs. 838). As a result, we are convinced that the LiM rate of 14.2% in initially diagnosed EC patients is closer to the real‐world value.

Next, we explored significant risk factors and independent prognostic factors for patients with LiM and constructed prediction models. It is generally accepted that an AUC value >0.7 is synonymous with an accurate prediction model.^15^ Herein, the AUC for each nomogram was >0.73, suggesting a satisfactory prediction accuracy.

Variables including age, gender, tumor site, tumor grade, tumor size, primary tumor, regional lymph node, and concurrent organ metastases were independently related to the incidence of LiM, with concurrent organ metastases and tumor site showing the highest discriminating power. In addition, age, tumor grade, histology, concurrent organ metastases and chemotherapy were significantly associated with survival, with chemotherapy possessing the highest discriminating power. Taken together, the above results are in accordance with previous studies.^9^, ^11^, ^14^

In terms of age at diagnosis, younger age was found to be a risk factor for developing LiM while being a protective factor for survival. Seemingly contradictory, these results were reasonable. Compared to older patients, tumors in younger patients were found to be more aggressive, and they were more likely to develop distant metastases.^16^ This phenomenon was reported in several solid tumors, including EC.^16^, ^17^, ^18^ Conversely, younger patients with distant metastases showed favorable survival outcomes due to their better treatment tolerance and fitness^.^ ^19^, ^20^

Regarding gender, our research revealed that men had a higher risk of LiM. There was no agreement on the influence of gender differences on DM. Few previous reports revealed that the male gender was associated with a higher incidence of DM^,^ ^18^ while several studies found no significant difference.^17^, ^21^, ^22^

Previous studies demonstrated that tumor grade, tumor size, T stage, and regional lymph node were associated with DM and survival outcomes,^19^, ^20^ which is consistent with our findings.

In terms of concurrent organ metastases, considerable evidence revealed that synchronous multiple organ metastases were common, and the number of metastatic organs was independently associated with survival.^13^, ^14^, ^23^, ^24^ Guo et al. found that the incidence of LuM in EC was associated with the number of extrapulmonary metastatic sites.^14^ Moreover, Cheng et al. reported that BrM was significantly related to BoM and LuM.^13^ Concerning survival, Chen et al. reported that mEC patients with isolated organ metastasis had a favorable prognosis compared to those with multiple organ metastases.^24^ Tang et al. found that CSS of mEC patients was negatively correlated with the number of metastatic sites,^12^ which is consistent with our findings.

In terms of tumor site location, we found that the incidence of LiM increased with the descending of tumor location, which was in line with previous reports.^8^, ^11^, ^23^ The following causes might explain this phenomenon: the blood of the lower esophagus is drained via the left gastric vein, which enters the liver through the portal vein.^11^ In addition, the predominant histology of lower esophageal cancer is AC, which is more prone to distant metastasis.^8^, ^25^ The above two factors might synergistically increase the incidence of LiM. In terms of histology, studies show that patients with AC are more likely to develop DM, especially LiM.^8^, ^25^ LiM in patients with SCC histology generally indicates a more advanced stage. Indeed, SCC was revealed to be a negative predictor of survival in EC patients with DM.^12^ In addition, concurrent organ metastases denote a terminal stage malignancy, leading to shortened survival and a higher risk of DM in other organs.^12^

Regardless of the histology, systemic therapy has been proven to be the most effective way to relieve symptoms, enhance the quality of life, and extend survival for mEC patients.^4^, ^26^ Janmaat et al. systemically reviewed 750 cases from five clinical trials, revealing that chemotherapy or targeted therapy could benefit OS compared to best supportive care,^27^ which is in line with our results. In this research, chemotherapy was found to be an essential prognostic factor with the highest discriminating power.

Nevertheless, this study has some limitations. First, retrospective studies are inherently biased. Moreover, although our predictive and prognostic models were built using a large sample size, we did not have an external validation cohort to confirm our results. Furthermore, we are unaware whether chemotherapy and radiotherapy were adopted sequentially or concurrently from data obtained from the SEER database.

In conclusion, as far as we are aware, the present study has the largest EC patients with LiM cohort to date. Herein, the incidence of LiM, OS and CSS in EC patients with LiM is described, and independent predictive factors for LiM and significant prognostic factors of OS and CSS in EC patients with LiM identified. More importantly, a risk prediction nomogram for LiM likelihood, a survival prediction nomogram for OS and a survival prediction nomogram for CSS were established and showed good accuracy. Collectively, we hope that our findings can act as a reference for clinicians and future research.

## CONFLICT OF INTEREST

The authors declare that there is no conflict of interest.

## Supporting information


**Supplementary Figure 1** (A) Calibration slope curve and (B) receiving operating characteristic (ROC) curve of liver metastases risk prediction model.

**Supplementary Figure 2** Calibration slope (CS) curve and receiving operating characteristic (ROC) curve of the model predicting overall survival (OS) and cancer‐specific survival (CSS) for esophageal patients with liver metastases: (A) CS curve of 6‐month OS, (B) CS curve of 6‐month CSS, (C) CS curve of 12‐month OS, (D) CS curve of 12‐month CSS, (E) CS curve of 18‐month OS, (F) CS curve of 18‐month CSS, (G) ROC curve of OS and (G) ROC curve of CSS.

**Supplementary Figure 3** Receiving operating characteristic (ROC) curve of the model based on traditional TNM staging predicting (A) overall survival (OS), and (B) cancer‐specific survival (CSS) for esophageal patients with liver metastases.
