# Supplementary figures and images for: The risk and prognostic factors for liver metastases in esophageal cancer patients: A large‐cohort based study
Source: Thorac Cancer. 2022 Sep 28;13(21):2960–9. doi: 10.1111/1759-7714.14642 (PMC9626357; doi:10.1111/1759-7714.14642)

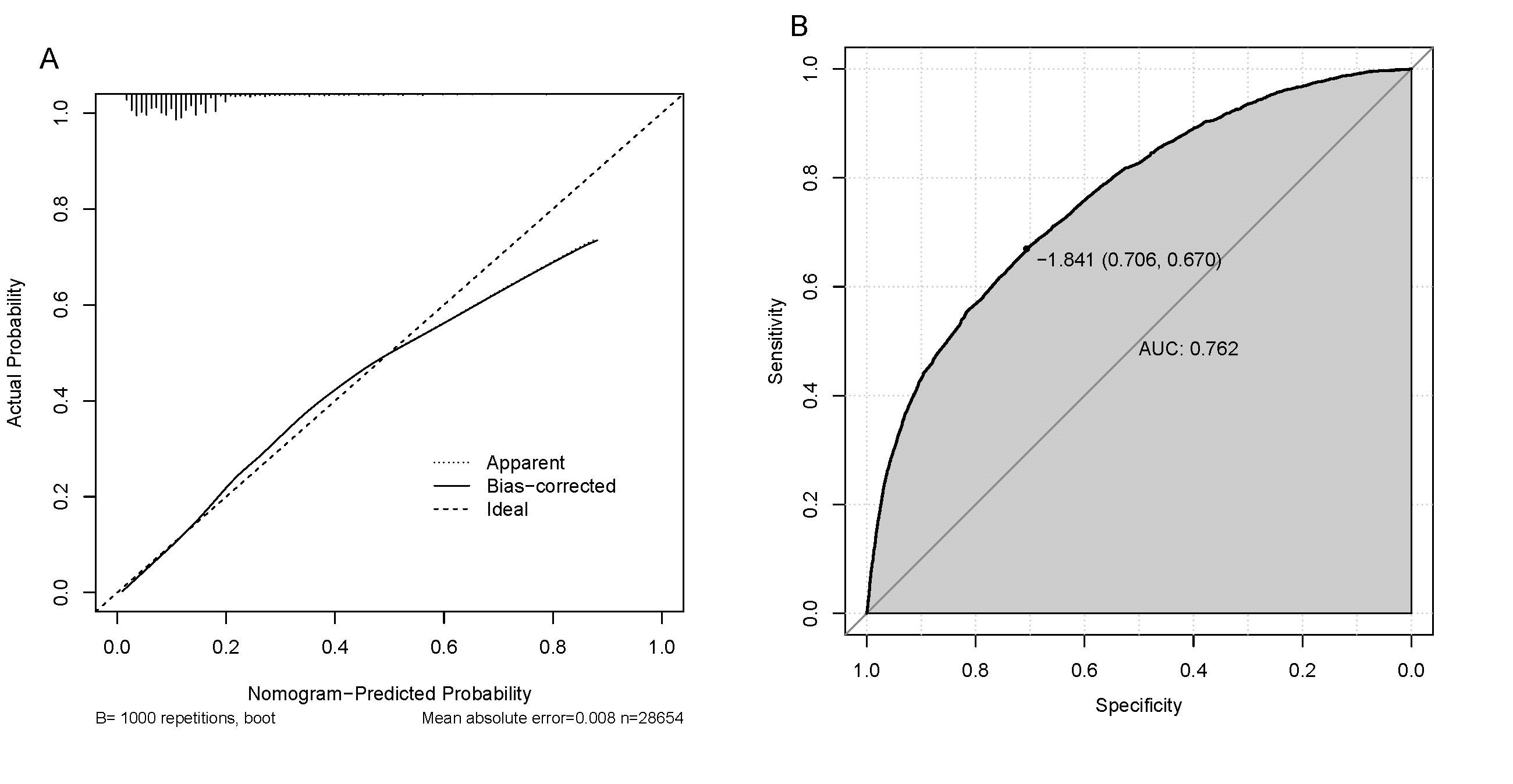

Supplement: Supplementary file 1 — Supplementary Figure 1 (A) Calibration slope curve and (B) receiving operating characteristic (ROC) curve of liver metastases risk prediction model. [file TCA-13-2960-s001.jpg]

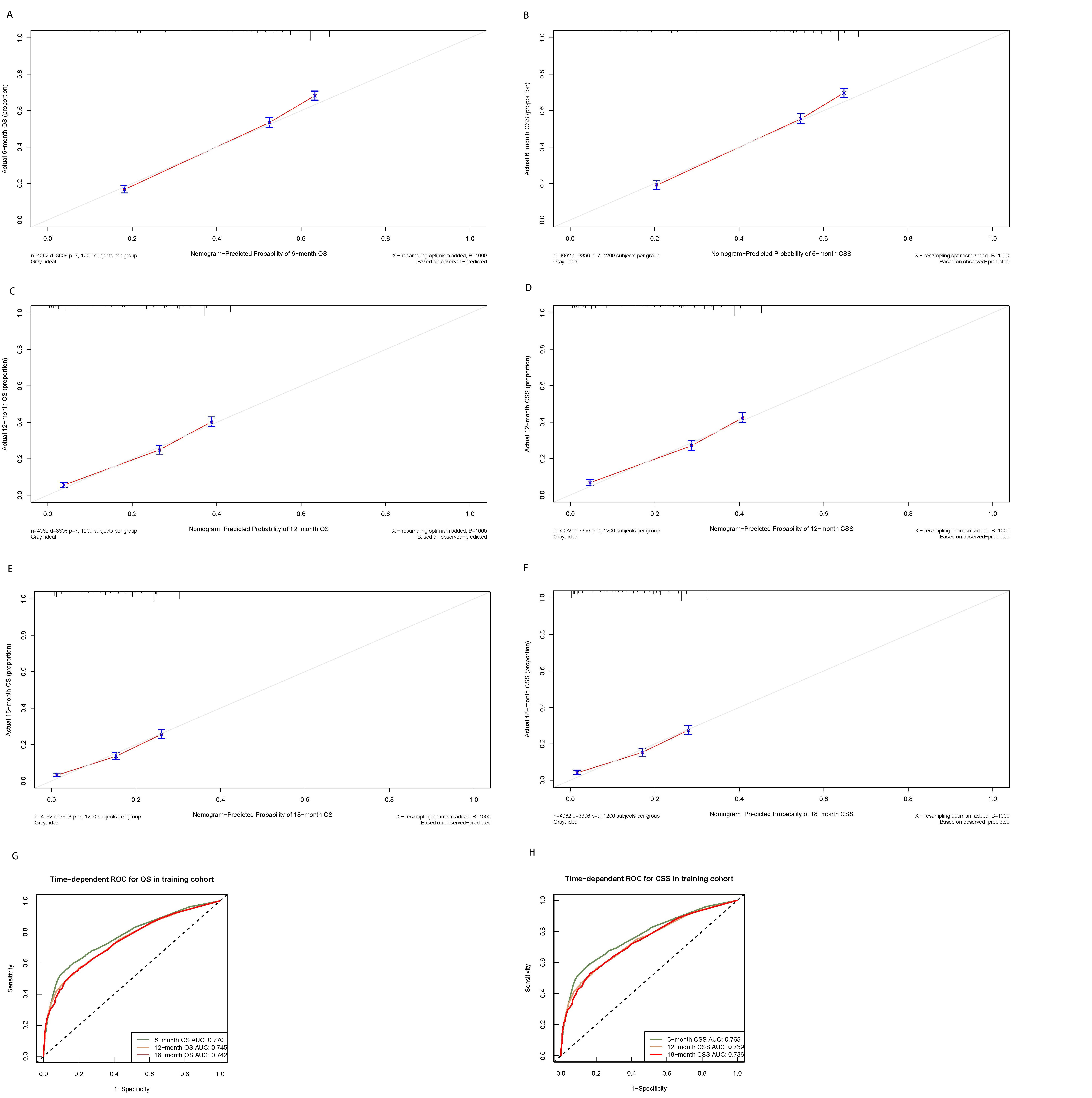

Supplement: Supplementary file 2 — Supplementary Figure 2 Calibration slope (CS) curve and receiving operating characteristic (ROC) curve of the model predicting overall survival (OS) and cancer‐specific survival (CSS) for esophageal patients with liver metastases: (A) CS curve of 6‐month OS, (B) CS curve of 6‐month CSS, (C) CS curve of 12‐month OS, (D) CS curve of 12‐month CSS, (E) CS curve of 18‐month OS, (F) CS curve of 18‐month CSS, (G) ROC curve of OS and (G) ROC curve of CSS. [file TCA-13-2960-s002.jpg]

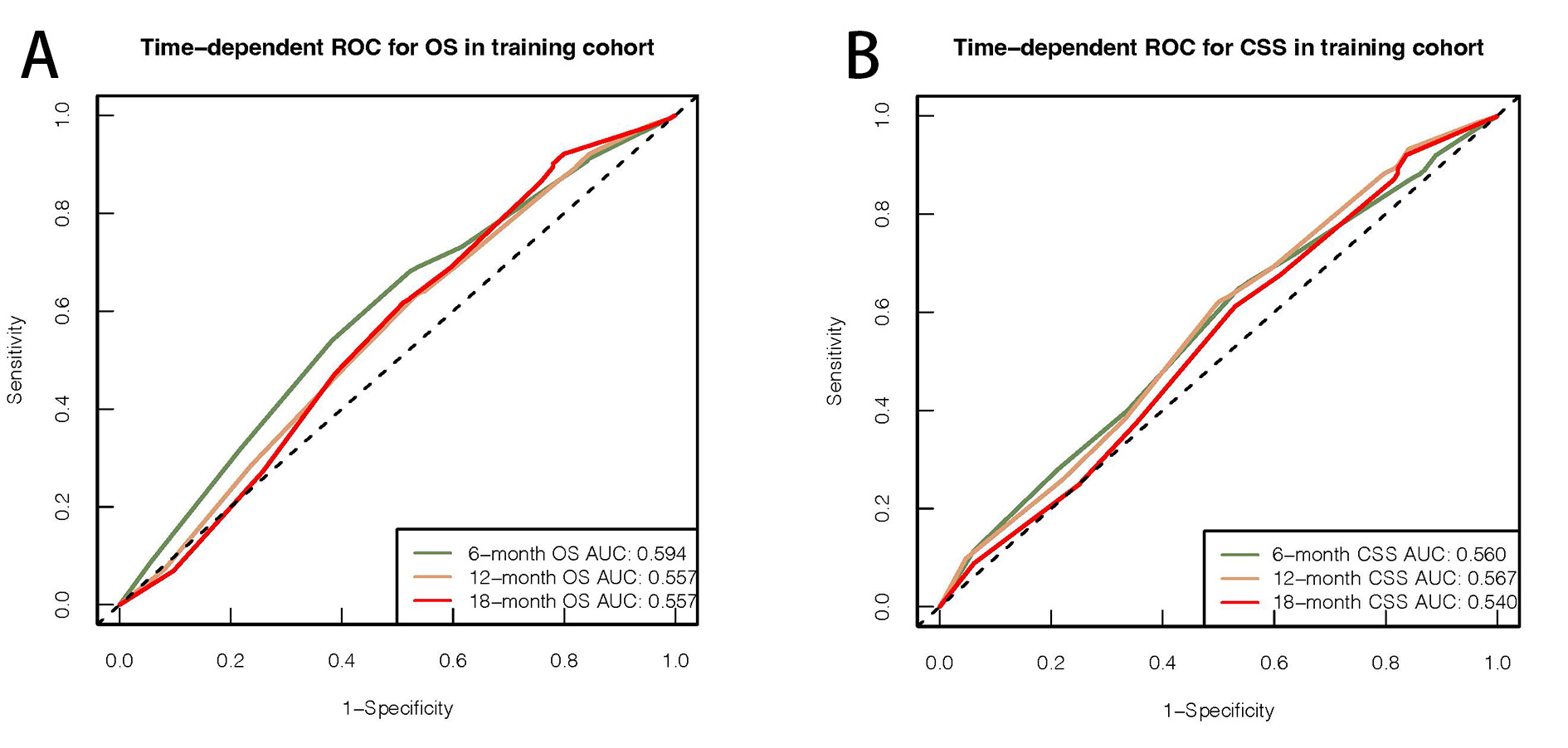

Supplement: Supplementary file 3 — Supplementary Figure 3 Receiving operating characteristic (ROC) curve of the model based on traditional TNM staging predicting (A) overall survival (OS), and (B) cancer‐specific survival (CSS) for esophageal patients with liver metastases. [file TCA-13-2960-s003.jpg]
